# Is there a right control condition in mHealth trials? A critical view on pain medicine

**DOI:** 10.1038/s41746-019-0184-z

**Published:** 2019-11-05

**Authors:** Janosch A. Priebe, Thomas R. Toelle

**Affiliations:** Center of Interdisciplinary Pain Medicine, Department of Neurology, Klinikum rechts der Isar (MRI), Technical University of Munich, Munich, Germany

**Keywords:** Clinical trials, Health services

## Abstract

When mobile health (mHealth) applications (apps) are investigated, the question of the proper control condition arises. Normally, the randomized controlled trial (RCT) is seen as the gold standard when testing efficacy of clinical interventions. Yet, mHealth apps rarely comprise innovative treatments but rather provide established treatments digitally. The classical RCT utilizing a placebo or waiting group condition may not always be the suitable methodology, since non-treatment is not appropriate if a disease urges treatment and the development of chronic disease needs to be prevented. The present commentary discusses conceivable control conditions in mHealth trials and illustrates their limitations.

## The randomized controlled trial in mHealth research

Since mHealth applications (apps) are finding their way into medical reality the apps also have to prove their efficacy in clinical trials.^1–3^ Usually, the randomized controlled trial (RCT) is seen as the gold standard in clinical research, when the superiority of an intervention compared to other treatments or placebos ought to be shown.^4^ For example, if a new chemotherapy is tested against an established regimen, patients are randomized to either the test group (new chemo) or the control group (established regimen). After having completed the treatments, the groups are compared regarding a primary outcome, like mean survival without recurrence.

Thereby, randomization is not a concept for clinical trials particularly. It is applied in non-clinical trials or experiments as well. The objective of the assignments to the groups by chance (randomization) is to control for known and unknown variables, which may disturb the effect of the independent variable (treatment) on the dependent variable (outcome). By controlling factors of potential unwanted influence internal validity and primary variance are maximized.^5^ By randomization, the expected impact of those variables should be equal in both the intervention and the control group (identical expected values). For sure, even when randomization is applied carefully biases may occur, which may result from non-blinded investigators or participants as well as statistical artefacts when random assignment does not lead to comparable expected values in both groups.^5^ In spite of those shortcomings, the RCT has been the flagship of clinical trials.^4^

When investigating mHealth apps, however, the RCT methodology may come to its limit. In the present commentary we focus on the control condition in mHealth trials and discuss potential challenges in selection of the proper control condition exemplarily for mHealth apps targeting low back pain.

## Providing established treatments digitally

Why is the control condition in mHealth trials so critical? Medical apps are rarely completely new treatments but rather aim to provide established treatments digitally and therefore in a broader range at any time at any place and more cost-effective.^1^ They may further be a promising tool for realizing guideline-conform treatments.

mHealth apps seem to be especially useful for pain disorders. Physical exercise elements, relaxation techniques, and educational units play an important role in treatment of pain disorders like migraine, back pain or fibromyalgia.^6^ Elements facilitating patients’ empowerment can be easily provided via apps,^1^ for example exercise videos including movement feedback, mindfulness instructions, education or symptom diaries, while interactive opportunities may increase adherence.

A closer look at the treatment of unspecific low back pain, i.e., lumbar back pain without medical signs of a specific underlying cause, allows deeper insights into the underlying problems. Yet, the conditions in other pain disorders are by no means different; therefore, our considerations can be applied to a broad range of pain disorders.

Although guidelines are available and conservative treatment focusing on patients’ empowerment is clearly recommended, treatment reality is unstructured and fragmented.^7^ Even though there is no evidence for long-term efficacy, surgery, and pharmacological treatment still prevail in treatment of back pain.^7^

## Multidisciplinary therapy in low back pain

The gold standard for the treatment of unspecific low back pain is the multidisciplinary therapy, which combines physical (physiotherapy) and psychological exercises (for example mindfulness) as well as psychoeducation.^8^ It is obvious that providing such treatments in broad ranges is hard to achieve because of the tremendous costs of day hospital treatments and the limited treatment opportunities. Therefore, for the treatment of unspecific low back pain, mHealth apps may be a cost-effective opportunity to provide multidisciplinary elements in a broader range to more patients.^9^

For sure, the efficacy of mHealth apps must be shown in clinical studies—and here one question arises: What is the proper control group in a clinical trial investigating mHealth apps.

Three alternatives are conceivable:

1. mHealth app vs. waiting group approach
  On the first glance, comparing a group using the mHealth app with a group without any treatment would be a promising approach. Yet, in the case of low back pain, two reasons contradict such an approach. First, there is good evidence that in the acute (up to 6 weeks) and subacute (6–12 weeks) stage of low back pain, immediate treatment is recommended in order to maximally prevent the development of chronic back pain in patients at risk.^10^ Therefore, a waiting group is ethically hardly acceptable. Second, since mHealth apps are not an innovative treatment by themselves but rather a tool to provide evidence-based treatment digitally in a broader range, a potential superiority to a waiting group would not be a striking finding.
2. mHealth app vs. guideline-conform face-to-face treatment approach
  Comparing the efficacy of an app providing guideline-conform treatment digitally to patients receiving face-to-face guideline-conform treatment may be an alternative. Yet, at best, non-inferiority of the app is expectable due to personal factors of the professional(s) providing the treatment (non-inferiority trial). In other words: Why should an app be the better physician? Furthermore, since guideline-conform face-to-face treatment de facto only takes place in day hospitals and not in the outpatient setting the patients receiving multidisciplinary pain treatment are serious cases who clearly differ from mHealth app users regarding symptom load. Treating serious cases solely by mHealth apps in a trial may again lead to an ethical problem.
3. mHealth app vs. regular treatment approach
  Given the preceding reasoning, the most promising approach may be comparing outcomes in patients using an mHealth app with patients receiving “regular treatment” in an outpatient setting. This approach investigates exactly the gap in the treatment reality mHealth apps may be able to close. Since guideline-conform treatment opportunities are rare and expensive and regular treatment often not oriented to guidelines,^7^ mHealth apps may be able to provide cost-effective guideline-conform treatment to those patients who demand effective treatment, but who, however, are not candidates for the day hospital approach.

## App-physician competition bias

Focusing on alternative 3, a methodological challenge arises: What is regular treatment and—even more problematic—how can it be operationalized. On the first glance, recruiting general physicians and instructing them: “Treat your back pain patients, like you always do” and then comparing those patients to “app-patients” seems to be the intuitive approach. But does this “Treat your back pain patients, like you always do” really reflects reality? If a physician is aware that he participates in a study and runs against an app, motivational factors may lead him to collect information about back pain, the guidelines etc. which in consequence leads to biased treatment with a higher quality than usual and just not “like you always do”. We call this the “app-physician competition bias”. In this setting, the app does not run against regular treatment but—at least in part—against guideline-conform face-to-face treatment, which is not the actual standard.^7^ And again—why should an app be the better physician?

## Innovative study designs

In line with these considerations, innovative study designs must be developed for mHealth app research. Investigating regular treatment seems only possible without the physician in the control group being aware of the study. This may only be doable by retrospective assessments of patients having been treated against back pain. Basically, such an approach may lead to extremely biased data, which are not comparable to RCTs regarding data quality. Yet, these weaknesses of retrospective data analyses may not count for mHealth app research as much as they do in the regular trial setting.

Compared to face-to-face pharmacological and non-pharmacological interventions much more data are available when mHealth apps are used. In these electronic tools the log-in files provide a wealth of data—given that the user agrees on their usage. In addition to symptom diaries—for example pain ratings in an app for low back pain—adherence to the programme can be determined precisely by analyzing user data (frequency of use, duration of use, information about completed elements of a programme). Since all those data are collected simultaneously with treatment, recall and other biases are prevented which in consequence significantly increases validity and value of retrospective analyses.^11^ These considerations are by far not new. They are in line with the concepts of Ecological Momentary Assessment^12^ and the micro-randomized controlled trial,^13^ which are suitable for evaluating behavioral changes in apps for cessation of bad habits, like smoking or passive lifestyle. Yet, causal relationships are still hard to prove by such non-controlled investigations especially because a comparably extensive dataset is not available for the retrospectively assessed control group having received regular treatment. In consequence, such approaches do not suffice in clinical research.

Despite these limitations we consider analyzing retrospective data of app users and especially analyzing treatment costs, which may be lower when guideline-oriented digital interventions replace non-evidence-based regular treatment, should be considered. In addition to “clean” RCTs^1–3^ these approaches may contribute to reducing the tensions between ethical and methodological issues.^12,13^ Furthermore, retrospective analyses may provide hints for conducting subsequent “clean” clinical trials. Yet, in the end, the retrospective approach will not be able to supersede between-group comparisons in clinical mHealth research (RCT).

We are well aware that our commentary exemplarily discusses the control group issue with major reference to low back pain apps. One may argue that this focus may limit our conclusions. Yet, we consider that our contemplations may also count for apps targeting other (pain) disorders by digitizing established treatments when their efficacy has to be shown in clinical studies.
